# Silent Intruders: A Dual Case Report of Retained Foreign Bodies in the Breasts

**DOI:** 10.7759/cureus.106208

**Published:** 2026-03-31

**Authors:** Radha Sarawagi, Monisha Kaliyan, Meena Kumari, Akarsh Singh, Swagata Bramhachari

**Affiliations:** 1 Department of Radiodiagnosis and Imaging, All India Institute of Medical Sciences, Bhopal, Bhopal, IND; 2 Department of General Surgery, All India Institute of Medical Sciences, Bhopal, Bhopal, IND

**Keywords:** breast abscess, breast foreign body, foreign body retention, gossypiboma, needles, retained surgical sponge

## Abstract

Foreign bodies in the breast are quite uncommon and can mimic both benign and malignant pathology on imaging. They may result from prior surgical interventions or trauma and have varied presentations. They also increase the need for multi-modality imaging to confirm the diagnosis, as they are less suspected. Awareness of this entity is important to avoid misdiagnosis and unnecessary aggressive intervention.

We present two interesting cases of foreign bodies in the breast parenchyma with distinct aetiologies, highlighting the different imaging presentations. This case report emphasises the significance of accurate imaging interpretation and appropriate imaging modalities for diagnosis.

## Introduction

Foreign bodies retained within the breast are extremely uncommon, accounting for merely 0.7% of unintended retained items within the human body. Most of these cases arise from surgical procedures and medical interventions [1]. The initial documentation of a case of gossypiboma was made by Wilson in 1884. The actual incidence of such cases is not well established because of legal and ethical concerns. Gossypibomas can manifest at any point, ranging from the immediate postoperative period to many years later [2]. The time of diagnosis for such cases is based on the inflammatory response triggered, post-surgical imaging and often due to a sensation of mass, produced by the retained foreign bodies [3].

Because of the unfamiliarity of this entity in the breast and its potential to mimic malignancy or granulomatous disease, awareness of its imaging appearances is essential for prompt diagnosis and appropriate management. Here, we present two unusual cases of foreign bodies retained in the breast with distinct etiologies and imaging features.

## Case presentation

Case one

A 40-year-old female patient presented with a one-month history of a lump in the left breast. The onset was insidious with gradual progression. The lump was associated with mild, intermittent, non-radiating, and pricking type of pain. She also reported foul-smelling purulent discharge from the left breast for a month, which was mild in quantity and intermittent in nature. Family history was significant for an unknown neck cancer in her grandmother.

On clinical examination, a 4x3 cm left breast lump was noted in the upper inner quadrant (10-12 o’clock) with hyperpigmented margins and surrounding induration. Over the lump, at the 11 o’clock position, 3 cm from the nipple areolar complex, a small sinus opening with purulent discharge was noted. There was no history of similar complaints in past and no known comorbidities. A clinical diagnosis of chronic abscess, with sinus secondary to granulomatous infection, was made.

**Figure 1:**
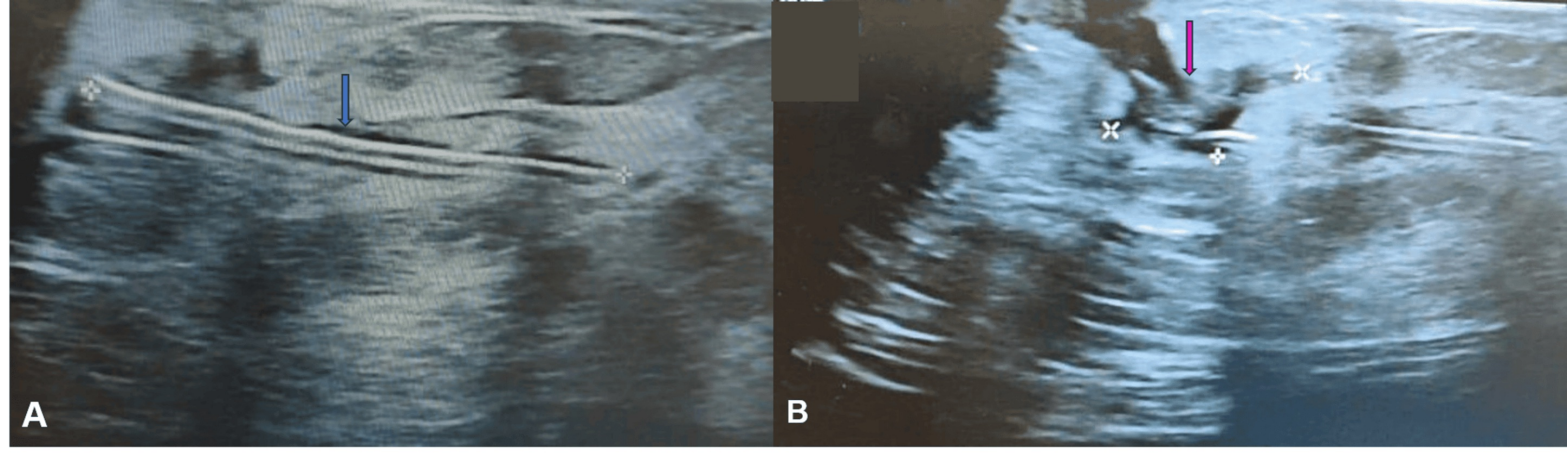
Ultrasonography images of the left breast (case one) (A) A linear hyperdense structure with central linear hypodensity consistent with a needle is seen (blue arrow). (B) An irregular hypodense area near the linear hyperdense needle with a tract-like extension into the skin surface is indicative of a collection (pink arrow).

Sonography of the left breast lump revealed a hypoechoic lesion with a central linear hyper-echoic structure giving a reverberation artifact. Findings were indicative of a metallic foreign body with surrounding granulation tissue and a small collection. On further evaluation, similar multiple linear hyper-echoic metallic foreign bodies measuring a maximum length of ~3.5 cm were noted scattered in the breast parenchyma, predominantly in the upper quadrant. No such foreign bodies were noted in the right breast (Figure 1).

**Figure 2:**
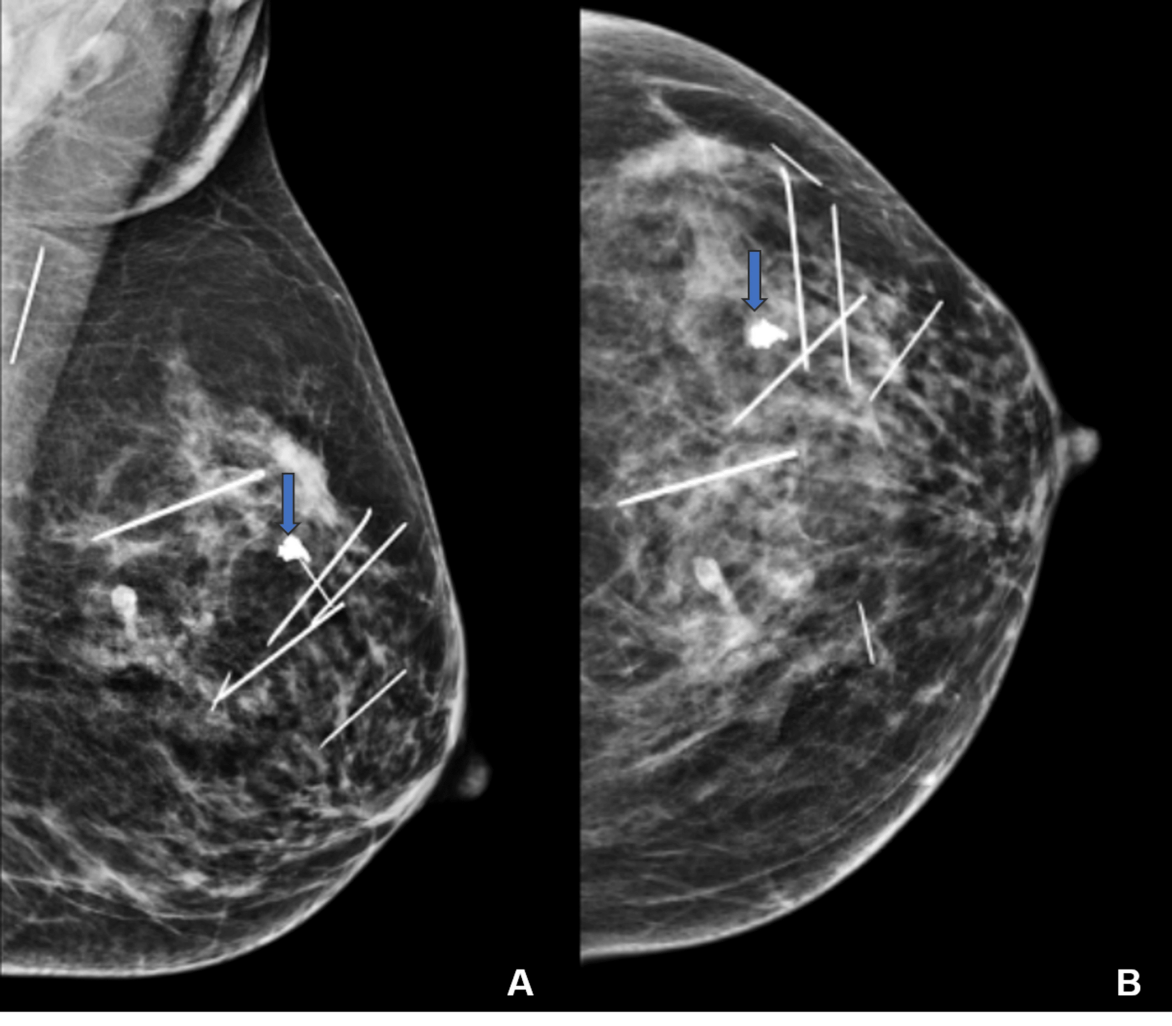
Mammography images of the left breast (case one) Mediolateral oblique (MLO) (A) and craniocaudal (CC) views (B) show multiple linear hyperdense metallic foreign bodies scattered within the breast parenchyma. A calcified involuting fibroadenoma is also noted (arrow).

There were a few simple cysts in the bilateral breasts. Mammography was performed, which revealed multiple linear hyperdense foreign bodies within the left breast parenchyma. One involuting fibroadenoma at 1 o’clock position in the left breast was also noted (Figure 2).

The patient consistently denied any prior intervention and self-insertion of foreign bodies. There was no history of accidental trauma, prior surgical procedures, or similar complaints in the past. Additionally, no history suggestive of substance abuse or underlying psychiatric illness was elicited. In view of the discrepancy between the clinical findings and the reported history, a psychiatric evaluation was sought. After careful exclusion of other medical and psychiatric conditions, including malingering and somatic symptom disorder, a diagnosis of factitious disorder was established.

Pre-operative marking of the position of a few foreign bodies was done. The patient underwent removal of the foreign bodies from the left breast via peri-areolar and lateral mammary incisions. Multiple foreign bodies were retrieved, including intact sewing needles and fragmented portions of hypodermic needles under C-arm and ultrasonography guidance. Complete removal of the foreign bodies was confirmed with the C-arm. The surgery and postoperative period were uneventful.

Case two

A 45-year-old female patient presented with a complaint of recurrent pus discharge from the left breast for 15 months. The patient had a history of incision and drainage for the left breast abscess in a private hospital, following which she had recurrent pus discharge from the incision site. She was started on antitubercular drugs for seven months based on suspicion of tubercular granulomatous mastitis. But the complaints did not resolve. The patient gave a history of disparity in breast size since puberty. On examination, the patient’s right breast was twice the size of the left. The patient had a peri-areolar scar with a sinus at 7 o’clock discharging minimal pus. Surrounding induration was present, with an ill-defined lump.

**Figure 3:**
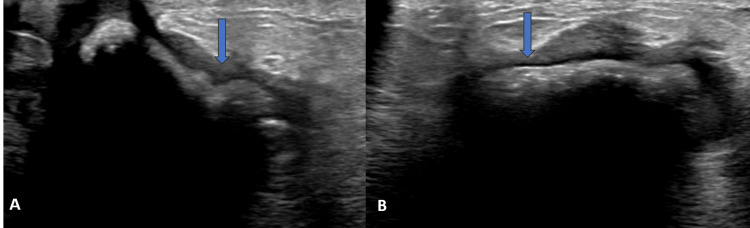
Ultra-sonography images (case two) (A) and (B) show a hypoechoic lesion with a central hyper-echoic wavy structure (arrow), producing dense posterior acoustic shadowing.

Sonography of the left breast lump revealed a hypoechoic lesion with a central hyper-echoic wavy structure giving dense posterior acoustic shadowing at the region of palpable lump (Figure 3).

**Figure 4:**
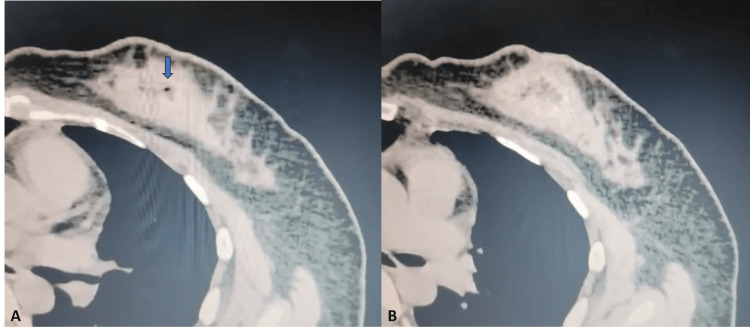
Computed tomography images (case two) (A) and (B) show an irregular soft tissue density mass in the central and inner quadrant of the left breast with central hypodensity and foci of air (blue arrow) giving a spongiform or honeycomb appearance, characteristic of retained surgical sponge (gossypiboma).

The possibility of retained surgical sponge (gossypiboma) was considered. Mammography could not be done due to technical reasons; hence, we proceeded with a limited-section computed tomography (CT) scan. Non-contrast CT scan revealed an irregular soft tissue density mass in the central and inner quadrant of the left breast with central hypodensity and foci of air giving a spongiform or honeycomb appearance characteristic of retained surgical sponge (gossypiboma) (Figure 4).

**Figure 5:**
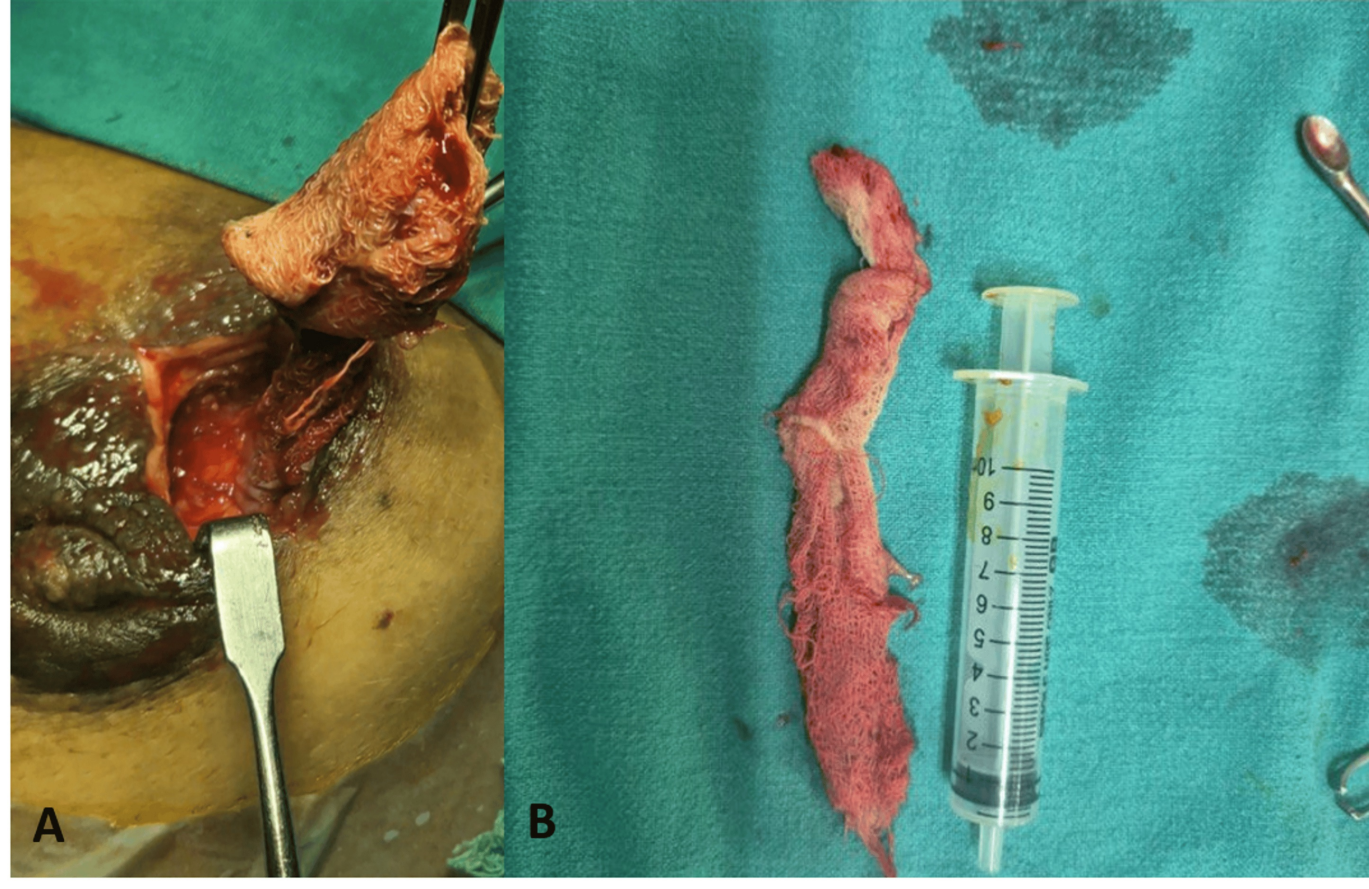
Intra-operative images (case two) (A) Intra-operative image shows the gauze piece being extracted from the breast. (B) Specimen image of the gossypiboma, which measured approximately 16-17 cm.

The patient underwent foreign body removal under local anesthesia with a peri-areolar incision, including the sinus. A surgical gauze measuring approximately 18 cm was found with abscess formation (Figure 5).

The excised sinus was sent for histopathological evaluation, which revealed an inflammatory lesion with a sinus tract and granulation. The postoperative period was uneventful. At a follow-up after three months, the patient was free of complaints.

## Discussion

Only a limited number of cases of foreign bodies in the breast have been reported in the literature. On mammography, radio-opaque foreign bodies such as metallic fragments or surgical clips appear as dense opacities. Non-radio-opaque materials may produce indirect signs such as irregular mass formation or architectural distortion, requiring the use of additional imaging modalities such as ultrasonography and computed tomography for accurate detection and localisation [4].

Gossypibomas are usually detected in the abdominal cavity. Very rarely, they are found in the extremities and superficial structures like the breast due to a lack of potential space to hold the surgical material [5]. They exhibit two distinct types of tissue reactions: an aseptic fibrotic response and an exudative inflammatory response. As the name suggests, in the aseptic type, there are inflammatory changes with eventual granuloma development. In the exudative response type, there is secondary bacterial contamination leading to abscess, sinus tract and fistula formation [2], as seen in our case.

The symptoms experienced by the patients depend on the location of the foreign body and the type of inflammatory reaction. Often, patients remain asymptomatic, as the body forms an aseptic granuloma. In cases of gossypiboma in the peritoneal cavity, the patient may present with obstructive features, secondary to adhesions caused by the foreign body reaction. They can also lead to abscess formation in the presence of secondary infections [6].

On a plain radiograph, it is difficult to localise the material, unless it has a radio-opaque marker. However, such markers are not routinely used [5]. In the absence of these markers, it becomes difficult to diagnose. They show a whirl-like appearance due to gas trapped in the sponge fibers on plain radiography [6]. Radiological diagnosis of a gossypiboma can be difficult due to the wide spectrum of imaging appearance. On mammography, gossypiboma may demonstrate a characteristic whorled or spongiform appearance [2,5]. On ultrasonography, it most commonly presents as a brightly echogenic lesion with a distinct posterior acoustic shadow, as observed in our case. Other patterns described in the literature include cystic, hypoechoic, and complex mass-like appearances, which are also relatively nonspecific [5].

On CT, the retained sponge may appear as a low-density heterogeneous mass, which on post-contrast can show peripheral rim enhancement. The presence of a gas bubble with a spongiform pattern is considered a characteristic feature on CT. Calcifications can also be present on the wall of the mass [7]. In cases where imaging features are strongly suggestive of malignancy in a suspected case of foreign body, pre-operative biopsy can be done to minimise the need for unnecessary surgery [8].

Other metallic foreign bodies, such as needles, are readily visible in plain radiographs as well as mammography, as in our case. Needles embedded in the subcutaneous tissues of the chest wall have the potential to migrate towards the intra-thoracic cavity, lung parenchyma, pericardium and in rare cases even to myocardium, leading to pneumothorax, hemothorax, intrapulmonary abscess, cardiac tamponade, endocarditis and even death [9].

The possibility of psychiatric conditions such as factitious disorder and malingering should be considered in cases of multiple foreign bodies. These cases can present with inconsistent histories and repeated health care visits. When clinical and imaging findings are incongruent or lack explanations, a self-inflicted cause should be considered. Thorough history taking and a multi-disciplinary approach, including psychiatric evaluation, is necessary for early diagnosis of such conditions. Early recognition is important to ensure appropriate psychiatric care.

Localisation of foreign bodies can be challenging during the surgery. Real-time image guidance plays a crucial role in the accurate localisation and safe removal of foreign bodies. It helps in accurate incision planning and minimally invasive removal, reducing tissue trauma and operative time. Sonography is the most used modality for superficial foreign bodies due to its ease of use. There is an additional advantage of avoiding ionising radiation exposure to the operating room personnel [9]. Fluoroscopy is useful for radiopaque foreign bodies, such as a needle. In our case, we have combined both intra-operative ultrasonography and C-arm, and removed all the needles without causing significant trauma to the breast tissue. At the end of the surgery, thorough examination of the surgical field for surgical sponge or gauze can minimise the chances of gossypiboma [10].

## Conclusions

Although uncommon, foreign bodies within the breast should be included in the differential diagnosis of breast lumps, particularly in patients with a history of surgery or implants. Awareness of the imaging appearances across various modalities enables prompt and accurate diagnosis, appropriate management, and helps prevent misinterpretation as a malignancy or granulomatous condition. While many cases remain asymptomatic, long-standing foreign bodies may present as palpable masses or abscesses. Early identification is important to prevent potentially serious complications. In instances involving deliberate needle insertion, psychiatric assessment is recommended to address the underlying factors and reduce patient morbidity.

In this report, correlation of radiological findings with detailed patient history proved essential in reaching the correct diagnosis and treatment planning. These cases show the importance of a high suspicion, particularly when imaging findings are atypical or when they do not indicate common breast pathologies. A multidisciplinary approach involving radiologists, surgeons, and mental health professionals can significantly improve diagnostic accuracy and overall patient care.
